# Supplementary material for: Streamlined and Abundant Bacterioplankton Thrive in Functional Cohorts
Source: mSystems. 2020 Sep 29;5(5):e00316-20. doi: 10.1128/mSystems.00316-20 (PMC7527133; doi:10.1128/mSystems.00316-20)
Supplement: TABLE S1 [file mSystems.00316-20-st001.pdf]

	TS	MC
No. OTUs	216	100
n_{eff}	22	3
sparcity	49	85
phyla assort.	0.35	0.213
class assort.	0.29	0.407
order assort.	0.29	0.409
family assort.	0.27	0.423
genus assort.	0.26	0.354
species assort.	-0.01	-0.015
