# Supplementary material for: Streamlined and Abundant Bacterioplankton Thrive in Functional Cohorts
Source: mSystems. 2020 Sep 29;5(5):e00316-20. doi: 10.1128/mSystems.00316-20 (PMC7527133; doi:10.1128/mSystems.00316-20)
Supplement: TABLE S2 [file mSystems.00316-20-st002.pdf]

	calculation	r	No. OTUs	p val	Test of:
All network OTUs	log(sum +1) TS vs log(sum +1) MC	-0.25	299	0.00	total abundance vs total abundance
	log(av +1) TS vs log(av +1) MC	0.58	299	0.00	average abundance vs average abundance
	log(av +1) TS vs % of MC	0.29	299	0.00	average abundance vs prevalence
	% TS vs % of MC	-0.06	299	0.27	prevalence vs prevalence
Nanopelagicales OTUs	log(sum +1) TS vs log(sum +1) MC	0.11	31	0.54	total abundance vs total abundance
	log(av +1) TS vs log(av +1) MC	0.33	31	0.07	average abundance vs average abundance
	log(av +1) TS vs % of MC	0.41	31	0.02	average abundance vs prevalence
	% TS vs % of MC	0.10	31	0.61	prevalence vs prevalence
Ca. Fonsibacter OTUs	log(sum +1) TS vs log(sum +1) MC	0.32	11	0.34	total abundance vs total abundance
	log(av +1) TS vs log(av +1) MC	-0.06	11	0.87	average abundance vs average abundance
	log(av +1) TS vs % of MC	-0.11	11	0.76	average abundance vs prevalence
	% TS vs % of MC	0.82	11	0.00	prevalence vs prevalence
